# Predicting self-reported research misconduct and questionable research practices in university students using an augmented Theory of Planned Behavior

**DOI:** 10.3389/fpsyg.2015.00535

**Published:** 2015-04-30

**Authors:** Camilla J. Rajah-Kanagasabai, Lynne D. Roberts

**Affiliations:** School of Psychology and Speech Pathology, Curtin University, Perth, WAAustralia

**Keywords:** research misconduct, data fabrication, data falsification, academic integrity, Theory of Planned Behavior, descriptive norms, justifications, questionable research practices

## Abstract

This study examined the utility of the Theory of Planned Behavior model, augmented by descriptive norms and justifications, for predicting self-reported research misconduct and questionable research practices in university students. A convenience sample of 205 research active Western Australian university students (47 male, 158 female, ages 18–53 years, *M* = 22, SD = 4.78) completed an online survey. There was a low level of engagement in research misconduct, with approximately one in seven students reporting data fabrication and one in eight data falsification. Path analysis and model testing in LISREL supported a parsimonious two step mediation model, providing good fit to the data. After controlling for social desirability, the effect of attitudes, subjective norms, descriptive norms and perceived behavioral control on student engagement in research misconduct and questionable research practices was mediated by justifications and then intention. This revised augmented model accounted for a substantial 40.8% of the variance in student engagement in research misconduct and questionable research practices, demonstrating its predictive utility. The model can be used to target interventions aimed at reducing student engagement in research misconduct and questionable research practices.

## Introduction

Academic integrity is vital to the foundation of the academic community and its credibility (McCabe and Trevino, 1993; McCabe et al., 2008). There are two types of dishonest misconduct that threaten academic integrity: academic misconduct (cheating, deception, and corruption; Mavrinac et al., 2010) and research misconduct (fabrication, falsification, and plagiarism in proposing and conducting research or reporting results; National Health and Medical Research Council and Australian Research Council, 2007). The US Department of Health and Human Services Office of Research Integrity (2000, p. 1) further define fabrication as making up data or results and reporting them, and falsification as “manipulating research materials, processes or changing or omitting data." Questionable research practices, consisting of failing to obtain approval, not obtaining consent before conducting research, ignoring outliers, publishing *post hoc* analyses without explanation, and publishing articles using data that have not been collected legitimately or that have been reported elsewhere (Pimple, 2002; Gilbert and Denison, 2003; Martinson et al., 2005; Kumar, 2008; Rose, 2008; Bedian et al., 2010), also fall within the umbrella of research misconduct. While data fabrication and falsification are the more serious forms of research misconduct, questionable research practices potentially have a larger impact on research integrity as they are more widespread (Anderson et al., 2013).

A growing body of research has examined research misconduct in academic settings. The most common form of research misconduct, plagiarism, is the area of research misconduct that has received the most attention (e.g., Park, 2003; Bennett, 2005; Marsden et al., 2005; Pickard, 2006; Mavrinac et al., 2010; Ogilvie and Stewart, 2010). In comparison, limited research has addressed fabrication, falsification, and questionable research practices in academic settings, and these areas are the focus of this research.

Estimates of the prevalence of research misconduct and questionable research practices among researchers and academics range widely, depending upon the measure used. Only 20–30 cases are reported to the US National Science Foundation and Department of Health and Human Service each year, representing a rate of 1 case per 100,000 researchers (Steneck, 2006). Estimates based on journal articles retracted for fabrication or falsification provide higher prevalence rates, but vary according to the years and databases covered. Based on analysis of article retractions in journals indexed by PubMed, Claxton (2005) estimated research misconduct was detected in less than one case per 5,000 papers (0.02%). Working on the assumption that for every case detected up to 10 cases may go undetected, Claxton estimated that the actual rate of fraudulent papers may be as high as 0.2%. Across databases, Grieneisen and Zhang (2012) identified 4449 articles retracted between 1928 and 2011, reporting that 20% were retracted for research misconduct, with a further 42% retracted for questionable data or interpretation. In contrast, using only articles indexed in PubMed, Fang et al. (2012) reported that 43% of the 2,047 articles retracted were retracted for fraud or suspected fraud. Articles retracted for data fabrication and/or falsification, in comparison to articles retracted for error, are clustered in high impact journals, have more authors and the first author is more likely to have previous retractions (Steen, 2010). Across retraction studies, the incidence of retracted papers is consistently reported to be increasing over time (Steen, 2011; Fang et al., 2012; Grieneisen and Zhang, 2012).

Higher prevalence estimates again are obtained when using self-report methodologies. In a recent meta-analysis, Fanelli (2009) reported that ~2% of scientists admitted to fabrication, falsification or modification of data at least once, whereas approximately a third admitted to questionable research practices. Interestingly, participants reported higher rates of awareness of at least one other researcher engaging in the fabrication of data (14%) and questionable research practices (72%). Further, self-reports may underestimate the actual prevalence of research misconduct and questionable research practices. John et al. (2012) provided incentives for honest reporting combined with anonymous reporting, with US academic psychologist respondents self-admitted questionable research practices ranging from 4.5% (claiming results unaffected by demographic variables when unsure/know false) to 66.5% (failing to report all of a study’s dependent variables).

Research misconduct and questionable research practices by researchers and academics may have roots in practices developed while students, and may reach back as far as the undergraduate years. Studies that have explored fabrication, falsification or questionable research practices in student populations have generally used student samples from degrees in ‘hard sciences,’ such as biomedical science, where the ‘correct’ answers to laboratory experiments are already known, making results more likely to be falsified (Davidson et al., 2001). Davidson et al. (2001) reported that 40–75% of undergraduate students admitted to ‘almost always’ manipulating data in science labs. Similar figures have been reported for other samples of science undergraduates (Franklyn-Stokes and Newstead, 1995; Lawson et al., 1999/2000). In contrast, figures are much lower (approximately one in five) when sampling undergraduates more broadly across disciplines outside of the sciences (Brimble and Stevenson-Clarke, 2005; McCabe, 2005). Of particular concern, one in ten Ph.D. students report falsification and fabrication of data is acceptable (Hofman et al., 2013).

Students who engage in academically dishonest behavior at university are likely to engage in dishonest behavior in the workforce (Nonis and Swift, 2001; Graves, 2008), highlighting the importance of understanding and addressing research misconduct at the time it first emerges, in the undergraduate years.

In attempting to understand dishonest behavior a range of competing economic, criminological and psychological theories have been used. In summarizing the factors shaping dishonest behavior across contexts, Ariely (2012, Figure 6) highlights the role of rationalizations, conflicts of interest, creativity, engaging in the first dishonest act, ego-depletion, benefit to others, observing the dishonest behavior of others and culture. Within academic settings, a range of theoretical frameworks, such as the General Theory of Crime (Gottfredson and Hirshci, 1990), Social Learning Theory (Bandura, 1978), Techniques of Neutralization (Sykes and Matza, 1957), Multidimensional Ethics Theory (Yang, 2012b) and the Theory of Planned Behavior (Ajzen, 1985) have been successfully applied in understanding academic dishonesty, but little research has focused on predicting fabrication, falsification and questionable research practices in university students. Of these theories, the Theory of Planned Behavior has consistently had good explanatory power, explaining 33–48% of the variance in health, social, and economic behavior (Armitage and Conner, 2001) and may be usefully applied to predicting engagement in research misconduct and questionable research practices.

### Theory of Planned Behavior

The Theory of Planned Behavior posits that intention drives behavior, with attitudes toward the behavior and subjective norms influencing behavior through intention, and perceived behavioral control impacting behavior both directly and mediated through intention (Ajzen, 1991). Attitudes represent positive or negative beliefs about behavior and its consequences. If a behavior is judged positively, attitude increases intention to engage in that behavior. Subjective norms represent perceived pressure from others to engage in behavior, and increase intention to engage in the behavior. Perceived behavioral control represents the perceived difficulty in performing the behavior, with greater difficulty reducing both intention to engage in behavior and actual behavior. Attitudes, subjective norms and perceived behavioral control form intention to perform a behavior, which if strong enough, will result in engagement (Ajzen, 1991). Ideally behavior is measured at a later point in time than intention, however, previous research has indicated that past behavior can be used as a proxy for future behavior (Rise et al., 2010).

Whilst not previously used to predict engagement in research misconduct and questionable research practices, the Theory of Planned Behavior has been used to predict cheating by undergraduate students. An early study by Beck and Ajzen (1991) used the Theory of Planned Behavior to predict a range of dishonest actions, including cheating on a test or exam. The Theory of Planned Behavior explained 67% of the variance in cheating intention and 55% of the variance in cheating behavior. However, subjective norms was not a significant predictor of intention and perceived behavioral control was not a significant predictor of behavior. Stone et al. (2009, 2010) examined cheating by undergraduate business students. The Theory of Planned Behavior explained 21% and 36% of the variance in cheating intention and cheating behavior respectively (Stone et al., 2010). Alleyne and Phillips (2011) examined undergraduate students’ intention to cheat and lie, reporting that Theory of Planned Behavior variables accounted for 48% of intention to cheat and 29% of intention to lie (actual behavior was not measured). Harding et al. (2007) found general support for the Theory of Planned Behavior model in predicting undergraduate cheating, but perceived behavioral control was not a significant predictor of behavior. In a further study, Mayhew et al. (2009) reported that neither attitudes nor perceived behavioral control were significant predictors of intention or behavior when moral obligation was added to the Theory of Planned Behavior model.

### Extending the Theory of Planned Behavior Model

A major strength of the Theory of Planned Behavior is that variables can be added to the model to increase its explanatory power (Ajzen, 1985). Two variables of interest in predicting engagement in research misconduct and questionable research practices are descriptive norms and justifications.

Descriptive norms relate to what others actually do (Rivis and Sheeran, 2003). As such, they represent the individual’s perception of behavior by others, in contrast to the traditional injunctive conceptualization of subjective norms where the focus is on the individual’s perception of perceived pressure from others to engage in a particular behavior (Ajzen, 1991). The distinction has been described in terms of ‘what is’ (descriptive norms) versus ‘what ought’ (subjective norms; also known as injunctive norms, Cialdini et al., 1990) to be done (Forward, 2009). Behavior is influenced by whether injunctive or descriptive norms are salient within a particular setting (Cialdini et al., 1990; Kallgren et al., 2000). Behavior by in-group members invokes descriptive norms, while behavior by out-group members invokes injunctive norms (Gino et al., 2009). Behavior is also influenced by the extent to which actions violate the salient norm and the personal norms of the individual (Kallgren et al., 2000). While injunctive norms may influence behavior across settings, descriptive norms influence behavior only in settings where they are salient (Reno et al., 1993). In more recent reconceptualizations of the structure of the Theory of Planned Behavior predictor variables, Fishbein and colleagues (Fishbein, 2000; Fishbein and Yzer, 2003; Ajzen and Fishbein, 2005) have noted the need to include both injunctive and descriptive norms “in order to obtain a complete measure of subjective norm” (Ajzen and Fishbein, 2005, p. 199). However, this practice does not appear to have been routinely adopted, with some research indicating injunctive and subjective norms are conceptually distinct and differentially predict intention and behavior (Forward, 2009; Manning, 2009).

Meta-analytic findings provide further support for the addition of descriptive norms to the Theory of Planned Behavior model. Descriptive norms and intention are medium-to-strongly correlated (*r* = 0.44) and account for an additional 5% of the variance in intention across a range of behaviors, after controlling for attitudes, subjective norms and perceived behavioral control (Rivis and Sheeran, 2003). However, descriptive norms were not predictive of intention for all behaviors, with moderator analyses indicating descriptive norms are of most importance in predicting intention to engage in risk behaviors and with younger samples (Rivis and Sheeran, 2003). Research predicting student engagement in research misconduct and questionable research practices meets both these criteria. A further meta-analysis by Manning (2009) indicated that the relationship between descriptive norms and behavior is stronger than the relationship between subjective norms and behavior, and that in modeling the Theory of Planned Behavior there is a direct path from descriptive norms to behavior, but only a mediated path from subjective norms to behavior. Descriptive norms have previously been demonstrated to be significantly correlated with both intention to engage in academic misconduct (*r* = 0.37) and actual academic misconduct (*r* = 0.49; Stone et al., 2010^1^), further justifying their addition to the Theory of Planned Behavior model.

As behaviors such as engaging in academic and research misconduct are not based on honest errors of judgment, individuals need to justify their engagement in the behavior (Stone et al., 2009). The mismatch between beliefs and behavior creates cognitive dissonance (Festinger, 1957), a psychological state that creates discomfort to the individual and motivates change to reduce the dissonance. More specifically, the term ‘ethical dissonance’ is used to describe cognitive dissonance resulting from behaviors deviating from accepted social norms (Barkan et al., 2012; Shalvi et al., 2015). Dissonance can be resolved through changing beliefs, changing behavior, adding new attitudes consistent with the behavior, or devaluing the importance of the dissonance (Festinger, 1957). Justifications may act to reduce dissonance through devaluating the importance of the dissonance (Stone et al., 2009). Self-serving justifications may reduce ethical dissonance through redefining and excusing questionable behaviors prior to engagement, or through compensatory mechanisms following engagement. Whether pre- or post-behavior, justifications attenuate the threat to the moral self (Shalvi et al., 2015).

Possible justifications for engaging in academic misconduct and questionable research practices include perceptions of others engaging in academic misconduct, helping a friend, peer pressure, extenuating circumstances and fear of failure (Stone et al., 2009). Stone et al. (2009) argue that justifications are used by those who have already engaged in academic misconduct, and play a potentially mediating role between the Theory of Planned Behavior predictor variables of attitudes, subjective norms and perceived behavioral control and the outcome variable of academic misconduct. In their study examining students’ cheating behavior, Stone et al. (2009) reported that attitudes, subjective norms and perceived behavioral control accounted for 28% of the variance in justifications, which in turn was a significant predictor of cheating behavior. Justifications were strongly correlated with both intention (*r* = 0.60) and behavior (*r* = 0.54). As academic and research misconduct are related constructs, this study provides strong support for the augmentation of the Theory of Planned Behavior model with justifications in predicting student engagement in research misconduct and questionable research practices.

Demographic factors may also be important in understanding student engagement in research misconduct and questionable research practices. Factors that have been explored in relation to this type of dishonest behavior are age, gender, and year of study. Negative correlations between age and academic misconduct have been reported (Brimble and Stevenson-Clarke, 2005), but inconsistent results found in relation to gender (Davidson et al., 2001; Yang, 2012a). A higher prevalence of research misconduct has been observed in lower year students (Yang, 2012b). Additionally, social desirability is an important construct to measure in self-report studies exploring research misconduct (Jann et al., 2012) as research misconduct is widely considered to be an unethical practice (Arvidson, 2004) and may elicit socially desirable responses.

**FIGURE 1:**
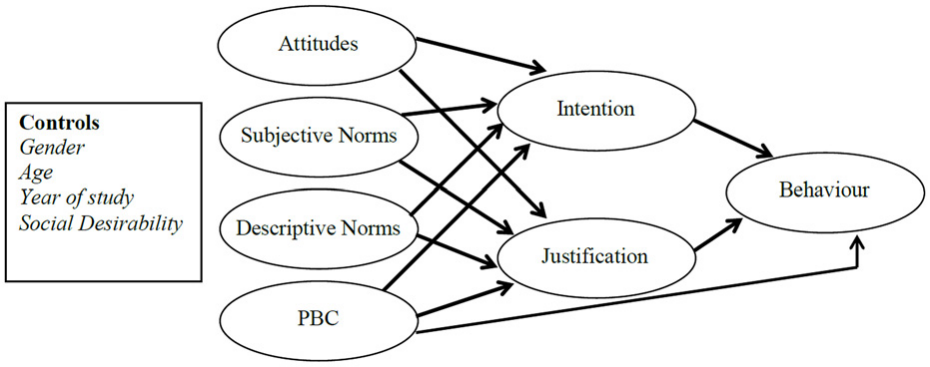
**Model of theory of planned behavior augmented by descriptive norms and justifications**.

In summary, there is limited research examining the predictors of student engagement in research misconduct and questionable research practices. The Theory of Planned Behavior is one model that may have utility in understanding these behaviors. Previous research that has examined the Theory of Planned Behavior in relation to academic integrity has mainly focused on cheating, but has demonstrated good explanatory power in some studies (Stone et al., 2009; Alleyne and Phillips, 2011). Drawing together previous disparate research on predictors of dishonest behavior into an integrated model applied to academic integrity, this study will examine the predictive utility of the Theory of Planned Behavior model augmented by descriptive norms and justifications (see **Figure 1**) in describing student engagement in research misconduct and questionable research practices. It is hypothesized that after controlling for demographic variables (age, gender, years of study) and social desirability, intention and justification will mediate the relationships between attitudes, subjective norms and descriptive norms with behavior (engaging in research misconduct and questionable research practices), and partially mediate the relationship between perceived behavioral control with behavior.

## Materials and Methods

### Research Design

This study used a self-report, correlational design to examine whether intention and justification (mediator variables) mediate the relationship between attitudes, subjective norms, descriptive norms, and perceived behavioral control (predictor variables) and student engagement in research misconduct and questionable research practices (criterion variable) while controlling for age, gender, years of study, and social desirability.

### Participants

A non-probability, convenience sample of Western Australian university students aged 18 years and older who had collected data or conducted research for an assignment or dissertation were recruited. The final sample consisted of 205 participants from five Western Australian universities (47 male, 158 female), aged between 18 and 53 years (*M* = 22, SD = 4.78). The majority of students sampled had a major or minor in Psychology (71.7%) and were from one university (84.8%). Years of completed study in university ranged from half a year to 9 years (*M* = 2.54, SD = 1.46). An a-priori power analysis (power 0.80, alpha 0.05) indicated that based on partial correlations of previous analyses (Stone et al., 2009), a sample size of 200 participants would be required to detect a ‘moderate’ mediation effect (Soper, 2013). The sample obtained exceeded this estimate and was deemed sufficient for testing mediation (Tabachnick and Fidell, 2007).

### Measures

An online questionnaire consisting of eight scales was developed using Qualtrics software. **Table 1** provides a summary of the measures, number of items, example items, response formats and Cronbach’s alpha for each measure. At the beginning of the survey, and at the top of most pages of the survey, the following definition of research misconduct was provided:

**Table 1:** Details of scale measures (*N* = 205).

Variable	Scale	No. of Items	Example Item (How responses were measured)	Scale range	α	Mean (SD)
Behavior	Adapted from Yang (2012a)	9^a^	How many times have you falsified results? (four point frequency scale – 1 = *never*, 2 = *one or two times*, 3 = *three to five times* and 4 = *six or more times*)	1–3	0.91	1.15 (0.29)
Attitudes	Adapted from Stone et al. (2009)	6^b^	It is always wrong to engage in research misconduct (five-point Likert scale – 1 = *strongly disagree* and 5 = *strongly agree)*	1–4	0.81	2.17 (0.63)
Subjective norms	Adapted from Beck and Ajzen (1991)	3	If I engaged in research misconduct, most people who are important to me would” (7-point Likert scale – 1 = *not care* and 7 = *disapprove*)	1–7	0.74	5.33 (1.47)
Descriptive norms	Adapted from Stone et al. (2009)	4^c^	**Quantity item** – Approximately what percentage of students do you think engage in some kind of research misconduct? (open response) **Frequency item** – How frequently do you think research misconduct occurs in classes at your university? (1 = *never*, 2 = *less than once a month*, 3 = *once a month*, 4 = *2–3 times a month*, 5 = *once a week*, 6 = *2–3 times a week* and 7 = *daily*)	0–100		26.46 (20.65)
Perceived behavioral control	Adapted from Stone et al. (2009)	4	It is easy to engage in research misconduct and not get caught (5-point Likert scale – 1 = *strongly disagree* and 5 = *strongly agree*)	1–5	0.89	2.83 (0.97)
Intention	Adapted from Yang (2012a)	9	How likely are you in the next year, to falsify results (5-point Likert scale – 1 = *very unlikely* and 5 = *very likely)*	1–4	0.91	1.51 (0.63)
Justifications	Adapted from Stone et al. (2009)	9	How likely are you to engage in research misconduct, because of laziness (5-point Likert scale – 1 = *very unlikely* and 5 = *very likely)*	1–4	0.92	1.96 (0.79)
Social Desirability	Adapted from Francis et al. (1992)	12^d^	Do you always practice what you preach? (dichotomous scale – 1 = *no* and 2 = *yes)*	1–2	0.71	1.66 (0.17)

*^a^Two items were removed due to low factor loadings; ^b^One item was removed due to low factor loading; ^c^This scale was replaced with a single item; ^d^One item was removed to increase scale reliability.*

Research Misconduct includes:
  Fabrication – making up data or results and reporting them
  Falsification – manipulating research materials or processes, or changing or omitting data
  Questionable research practices – failing to obtain approval, not obtaining consent before conducting research, ignoring outliers, publishing *post hoc* analyses without reporting it, or publishing articles using data that has not been collected legitimately or that has been reported elsewhere.

### Procedure

Ethics approval was received from Curtin University Human Research Ethics Committee. Participants were recruited on campus, from a psychology student participant pool and online through social networking sites. The recruiting materials directed potential participants to a Participant Information Sheet hosted on a university website and then linked to the online questionnaire. Consent was assumed upon submitting the questionnaire. Students recruited through the student participant pool were awarded points for participations and other students were provided with the opportunity to enter a draw to win a $50 Amazon.com gift voucher.

Data for 248 cases was downloaded from curtin.qualtrics.com into SPSS (version 21) for data preparation, and cleaning. Duplicate cases and cases with patterned responses or substantial missing data were removed, leaving 205 cases for analysis. A Missing Values Analysis indicated 0.38% missing data across the questionnaire. Little’s MCAR test indicated the data was not missing completely at random: χ^2^ (1053, *N* = 205) = 1173.68, *p* = 0.006. Expectation Maximization was used to replace missing values. Items were checked for outliers and unusual cases, and scale items were reverse coded where required. Descriptive norms item 3, “In the past year how many students do you think have engaged in research misconduct and have not been caught,” was excluded from further analyses due to wide variability in the types of responses yielded, including precise quantitative estimates (76.55%), vague qualitative estimates, such as “a few” (18.53%) and missing data (4.87%).

Confirmatory Factor Analysis was conducted in EQS 6.1to confirm the factor structure of scales in the augmented Theory of Planned Behavior model. Comparative fit indices, with recommended cut-offs from Kline (2011) were used to evaluate the fit of each scale. Based on poor fit statistics and identification of items with low loadings, the attitudes scale was reduced from six-items to five items and the behavior scale was reduced from nine-items to seven items. Goodness of fit statistics could not be computed for the Subjective norms and Descriptive norms scales, and for these measures Principal Axis Factoring supported one-factor solutions. A low Cronbach’s alpha of 0.16 and small positive correlations between items indicated the descriptive norms scale was unsuitable for use. Instead, the single item, “Approximately what percentage of students do you think engage in some kind of research misconduct?” was used to represent descriptive norms. Cronbach’s alpha was calculated for each of the measures (see **Table 1**). The 12-item original social desirability scale yielded a Cronbach’s alpha of 0.69. An examination of the questionnaire item-total statistics indicated an improved alpha of 0.71 if the item, “If you say you will do something, do you always keep your promise no matter how inconvenient it might be?” was deleted. This item was deleted, leaving an 11-item scale.

## Results

### Descriptive Statistics

There was a low level of engagement in the more serious forms of research misconduct. Analysis at the item level (**Table 2**) indicates that approximately one in seven students reported engaging in fabrication and one in eight students in falsification. The proportion of students engaging in questionable research practices varied by type of practice. In total, 39.5% of students admitting to engaging in at least one form of research misconduct (including questionable research practices) at least once.

**Table 2:** Percentage of participants self-reporting engaging in research misconduct.

Behavior	% Engaged in behavior
Claimed to conduct research that was not actually conducted	10.3
Reported research results without obtaining consent from peers	4.9
Claimed to use research materials that were not actually used	17.6
Fabricated information or research data	14.6
Falsified results	12.2
Concealed poor experiment or research data	16.6
Deliberately provided the wrong references	17.1
Deliberately ignored, concealed or distorted unfavorable research results claims	19.5
Provided references at the wrong place of the assignment	37.2

A summary of descriptive statistics for each measure is presented in **Table 1**. Descriptive norms, intention, justifications, social desirability, and behavior were positively skewed and subjective norms negatively skewed. Analyses were conducted with and without transformations of variables, however, as the results were approximately equivalent the results of the untransformed data are presented for ease of interpretation.

Age, gender, and years of study were not significantly associated with research misconduct behavior and were dropped as control variables. Only social desirability was significantly related to behavior and was retained as the sole control variable for further analyses.

### Testing the Augmented Theory of Planned Behavior Model

Prior to commencing analysis, assumptions underlying mediation (Baron and Kenny, 1986) were tested in the correlation matrix (**Table 3**). The criterion variable (behavior), mediators (intention and justification) and predictors (attitude, subjective norms, descriptive norms, perceived behavioral control) were significantly correlated, meeting the requirements for mediation testing. A partial correlation matrix was computed, to control for the effects of social desirability. Path analysis was conducted using LISREL software to enable the simultaneous assessment of all pathways in the model. The testing was conducted in stages. Fit statistics for each stage of testing are presented in **Table 4**.

**Table 3:** Pearson’s correlations between model and control variables.

	1	2	3	4	5	6	7	8	9	10	11	12
(1) Attitudes	1											
(2) SN	-0.42***	1										
(3) DN	0.02	-0.13	1									
(4) PBC	0.09	-0.06	0.17*	1								
(5) Intention	0.33***	-0.34***	0.25***	0.24**	1							
(6) Justification	0.32***	-0.37***	0.24***	0.28***	0.71***	1						
(7) Behavior	0.24**	-0.23**	0.30***	0.21**	0.63***	0.52***	1					
(8) SD	0.00	0.08	0.11	0.08	0.04	0.03	-0.16*	1				
(9) Gender	-0.10	0.04	0.10	-0.19**	-0.15*	-0.06	-0.03	0.10	1			
(10) Age	-0.18**	0.19**	-0.05	0.12	-0.14*	-0.19**	-0.12	0.06	0.02	1		
(11) Yrs of Stdy	-0.05	0.12	0.14*	0.22**	-0.13	-0.08	0.02	-0.08	0.10	0.28***	1	
(12) Und/Post	0.20	0.09	0.05	0.17*	-0.10	0.02	0.12	-0.08	0.03	0.14*	0.37***	1

SN, subjective norms; DN, descriptive norms; PBC, perceived behavioral conrol; SD, social desirability; Und/Post, undergraduate/postgraduate; Yrs of Stdy, years of study; *p < 0.05; **p < 0.01; ***p < 0.001.

**Table 4:** Fit indices for models tested.

Model testing	*X*^2^ sig	CFI	NNFI	SRMSR	RMSEA	AIC	BIC
*Recommended value*	*p* > 0.05	≥0.9^a^	≥0.9^a^	<0.1^b^	≤0.05^a^	lowest	lowest
Stage 1 Partially mediated model	*p*< 0.001	0.79	-3.47	0.10	0.69	1197	1287
Stage 2 Three pathways removed	*p*< 0.001	0.79	-0.08	0.10	0.34	1192	1271
Stage 3 Pathway from justification to intent added	*p* = 0.93	1.00	1.039	0.01	0.0	1095	1178
Stage 4 Revised model	*p* = 0.13	0.99	0.98	0.04	0.05	1097	1163

*X^2^, chi square test; CFI, comparative fit index; NNFI, non-normed fit index; SRMSR, standardized root mean square residual; RMSEA = root mean square error of approximation; AIC = Akaike information criterion; BIC = Bayesian information criterion; ^a^Benet-Martínez and Karakitapoglu-Aygün (2003); ^b^Marsh et al. (2004).*

In the first stage, a partial mediation model was tested. The direct pathways between attitudes and behavior and subjective norms and behavior were non-significant, consistent with the fully mediated relationship in the posited model. However, in contrast to the posited model, the direct pathway between perceived behavioral control and behavior was non-significant, indicating perceived behavioral control is fully mediated by intention and justifications. Also in contrast to the posited model, there was a significant direct pathway between descriptive norms and behavior, indicating a partially, rather than fully mediated relationship.

In the second stage, the model was rerun with the non-significant pathways between attitudes and behavior and subjective norms and behavior removed. All remaining pathways were significant. The predictor variables (attitudes, subjective norms, descriptive norms, and perceived behavioral control) accounted with 23.5% of the variance in intention and 25.6% of the variance in justifications. Intention, justifications and descriptive norms accounted for 38.7% of the variance in behavior. Modification indices indicated a pathway from justification to intent would improve model fit. This pathway is plausible as it is likely that viewing research misconduct behaviors as justifiable would precede the formation of intent to engage in those behaviors.

In the third stage, the pathway from justification to intent was added and the model rerun. With the pathway added, the four other predictors of intent (attitudes, subjective norms, descriptive norms and perceived behavioral control) were no longer significant, suggesting that a simplified model was required, with the relationship between the four predictors and intent fully mediated by justifications.

**FIGURE 2:**
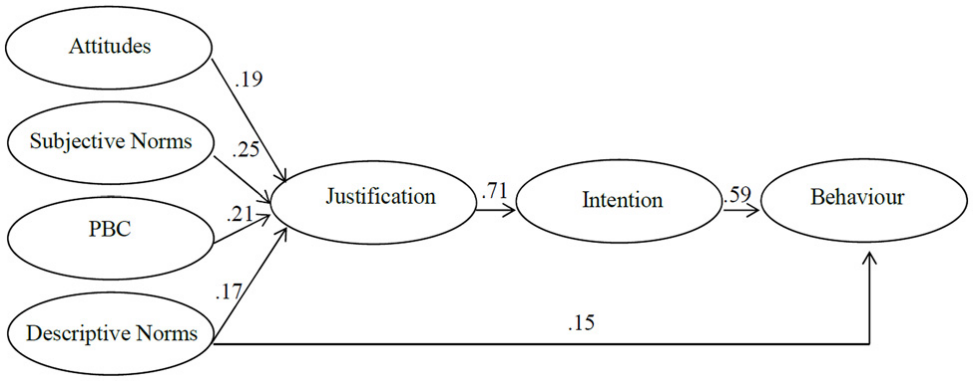
**Revised model of theory of planned behavior augmented by descriptive norms and justifications**.

In the fourth stage, this revised model (**Figure 2**) with the relationships between predictor variables and justification mediated by intent, was tested. This model accounted for 25.6% of the variance in justifications, 50.7% of the variance in intent and 40.8% of the variance in behavior. The Chi Square test was non-significant and fit statistics indicated good model fit to the data. While some fit statistics are superior for the model in the third stage of testing, the final revised model is preferred as it presents a more parsimonious model with good fit statistics and no non-significant pathways.

## Discussion

This research examined the Theory of Planned Behavior model, augmented by descriptive norms and justification, in predicting student engagement in research misconduct and questionable research practices. Model testing identified a parsimonious two step mediation model provided good fit to the data. The effect of predictor variables (attitudes, subjective norms, descriptive norms, and perceived behavioral control) on behavior was mediated by justifications, with justifications in turn mediated by intention. This revised augmented model accounted for a substantial 40.8% of the variance in student engagement in research misconduct and questionable research practices.

Examination of individual pathways indicates that attitudes, subjective norms, descriptive norms, and perceived behavioral control combined influence intent to engage in research misconduct and questionable research practices through informing the development of justifications (accounting for just over a quarter of the variance in justifications). Justifications accounted for more than half the variance in intent, highlighting the important role of justifications in intent to engage in research misconduct. This is consistent with previous research findings of the important role of justifications/rationalizations/neutralizations in shaping academic dishonesty (e.g., Haines et al., 1986; Labeff et al., 1990; Rettinger and Kramer, 2009; Meng et al., 2014).

As hypothesized, the effect of attitudes and subjective norms on behavior was fully mediated by justification and intention, although the effect of descriptive norms on behavior was only partially mediated. Contrary to the hypothesized partial mediation relationship, the effect of perceived behavioral control on behavior was fully mediated by justification and intention.

These results demonstrate the utility of the augmented Theory of Planned Behavior model in predicting student engagement in research misconduct and questionable research practices. The addition of justifications to the model helps explain the relationship between predictor variables and intent when predicting these dishonest behaviors. The results indicate that viewing research misconduct and questionable research practices positively, believing significant others to also view these positively, perceiving other students to be engaged in these dishonest behaviors and perceiving engaging in these behaviors as easy are associated with justifying engagement in research misconduct and questionable research practices, leading to greater intent and extent of involvement in research misconduct and questionable research practices. However, as this study is cross-sectional it is not possible to establish the causal direction of these findings. It is possible that, as proposed by Stone et al. (2009) in relation to academic misconduct, cognitive dissonance resulting from engagement in research misconduct and questionable research practices has resulted in individuals trivializing or amending their cognitions in order to reduce dissonance. The addition of descriptive norms increased the predictive ability of the Theory of Planned Behavior model, contributing directly to the prediction of student engagement in research misconduct and questionable research practices and indirectly through justifications. These findings are consistent with previous research findings indicating the importance of observing others’ dishonest behavior (Rettinger and Kramer, 2009) and support the utility of adding descriptive norms (Rivis and Sheeran, 2003; Forward, 2009; Stone et al., 2009, 2010; White et al., 2009) and justifications (Stone et al., 2009) to the Theory of Planned Behavior model.

Subjective and descriptive norms were differentially associated with intention, justifications and behavior. Subjective norms were more strongly associated with intention (*r* = -0.34) and justifications (*r* = -0.37) than behavior (*r =* -0.23), while descriptive norms were more strongly associated with behavior (*r* = 0.30) than intention (*r* = 0.25) or justifications (*r* = 0.24). While both types of norms were predictors of intention, only descriptive norms was predictive of behavior once other variables were controlled. These findings support Fishbein and colleagues’ recommendation to model both injunctive and descriptive norms within studies (Fishbein, 2000; Fishbein and Yzer, 2003; Ajzen and Fishbein, 2005), and are consistent with meta-analytic results indicating the relationship between descriptive norms and behavior is stronger than the relationship between subjective norms and behavior (Manning, 2009).

In this study ~40% of students admitting to engaging in at least one form of research misconduct at least once, with one in seven reporting engaging in data fabrication and one in seven engaging in falsifying results. Falling within the lower range of previous estimates of the prevalence of student research misconduct (Franklyn-Stokes and Newstead, 1995; Lawson et al., 1999/2000; Davidson et al., 2001; Brimble and Stevenson-Clarke, 2005; McCabe, 2005), these results confirm that engagement in research misconduct is not restricted to the ‘hard sciences,’ but is also present to some degree in other disciplines such as psychology.

The consistently reported student engagement in research misconduct and questionable research practices across studies highlights the need to address this type of dishonest behavior in undergraduate and postgraduate programs. The revised augmented Theory of Planned Behavior model increases our understanding of the routes to student engagement in research misconduct and questionable research practices and can be used to identify potential strategies to address these behaviors in universities. Attitudes were a significant predictor of justifications for engaging in research misconduct and questionable research practices. Explicit teaching in research methods courses about resultant harms from these behaviors may help foster a climate where research misconduct is viewed as unacceptable. For example, Boskovic et al. (2013) trialed discussion groups on research misconduct with Ph.D. students. The role of research mentors (Wocial, 1995; Wright et al., 2008; Kornfield, 2012) and supervisors (Mitchell and Carroll, 2008) in educating students about research integrity has also been stressed. However, it has been noted that mentors can exert both positive and negative influence in relation to research misconduct and questionable research practices (Anderson et al., 2007). Fostering a climate that values research integrity may also change subjective and descriptive norms over time.

A further avenue for reducing student engagement in research misconduct and questionable research practices is to directly address the justifications used to reduce ethical dissonance prior to engaging in these behaviors. Removing justifications for dishonest behavior reduces the likelihood of engaging in the behavior (Shalvi et al., 2012). Justifications may be addressed through increasing ethical salience and reducing ambiguity (Shalvi et al., 2015). Ethical salience can be increased through reference to moral codes and standards (Mazar et al., 2008). Further, previous research has indicated that signing a statement of honesty before self-reporting increases ethical salience and reduces dishonest reporting, in comparison to signing after self-reporting. Applying these findings to student research, students could be asked to sign a statement agreeing to engage in ethical research practices as outlined in relevant research ethics codes and guidelines prior to collecting or analyzing data. While completion and signing of ethics applications may serve this function for dissertation students, many lower level student research exercises do not have a requirement to complete and submit an ethics application. As part of the process of removing justifications, any ambiguity surrounding the acceptance of research misconduct and questionable research practices needs to be addressed. In particular, clarity is required on the body of behaviors referred to as ‘questionable research practices,’ with even the term itself suggesting ambiguity in whether or not these research practices are ethically acceptable. Teaching staff and research supervisors need to provide clear guidance to students on what is, and is not, acceptable research practice, providing applied disciplinary examples.

Perceived behavioral control was also a significant predictor of justifications, indicating that measures could be put in place to make it more difficult to engage in research misconduct and questionable research practices, or at least increase the perception that this type of dishonest behavior is likely to be identified. Procedures have already been developed to detect fabrication of data (Blasius and Thiessen, 2012), and these procedures have now been applied to detecting fabrication in honors dissertations (Allen et al., 2015). In the same way that students are currently required to submit work for plagiarism detection, it is possible in the future that students could be required to submit data-sets for fabrication detection.

### Limitations and Future Research

There are a number of limitations of this research that mean caution is required in the interpretation of these results. First, the descriptive norms measure had poor internal reliability, and an individual item providing ratio data was used in its place. This item was predictive of both justifications and behavior, indicating its importance and warranting further development of a descriptive norms measure for use in future research. Second, some variables exhibited non-normality and heteroscedasticity, violating assumptions underlying the analyses. However, analyses using transformed and untransformed data produced similar results, providing confidence in our findings. Third, self-report measures of past research misconduct and questionable research practices were used as a proxy for future behavior. While this is a common practice in Theory of Planned Behavior research (Armitage and Conner, 2001), future research separating the time of measurement of intention and behavior is recommended. This is particularly important when justifications are included in the model, as it has been argued that justifications may be made based on previous engagement in misconduct (Stone et al., 2009). Fourth, the reliance on self-report methods for all variables introduces the risk of common method variance/bias. However, recent *post hoc* research examining the effect of common method variance on Theory of Planned Behavior studies has indicated that common method variance is not a concern within this domain (Schaller et al., 2015). The reliance on self-report measures is also likely to have resulted in under-reporting of behavior (see John et al., 2012 for comparison of prevalence rates of questionable research practices with and without incentives for honesty in responding). Despite this, self-reports of engaging in research misconduct and questionable research practices provide a useful indicator of these behaviors. Previous research has demonstrated associations between self-reports of dishonest behavior and actual engagement in dishonest behaviors (Halevy et al., 2014), increasing our confidence in their use as proxies for actual behaviors. Finally, the majority of students in this study were psychology students from one university, limiting the generalizability of these findings to other academic settings. We recommend future research is based on larger samples across disciplines and universities, enabling a stronger test of the hypotheses. The actual and perceived seriousness and consequences of research misconduct and questionable research practices may vary according to student level and type of research project (e.g., assignment versus dissertation) and larger samples will enable an assessment of both the prevalence of these behaviors and the validity of the model by year group.

## Conclusion

In this research the Theory of Planned Behavior model, augmented by descriptive norms and justification, was used to predict student engagement in research misconduct and questionable research practices. The results support a two-step mediation model, where the effect of attitudes, subjective norms, descriptive norms and perceived behavioral control on behavior is mediated first by justifications, and then intention. The model has good utility, able to account for 40% of the variance of student engagement in research misconduct and questionable research practices.

## Conflict of Interest Statement

The authors declare that the research was conducted in the absence of any commercial or financial relationships that could be construed as a potential conflict of interest.

## Footnotes

1 Stone et al. (2010) labeled their normative measure ‘Subjective norm,’ but the items all reflect descriptive norms.
